# Chronic low back pain is associated with a reduction in lumbar movement – a prospective cohort study

**DOI:** 10.1038/s41598-025-04851-2

**Published:** 2025-06-05

**Authors:** Friederike Schömig, Matthias Pumberger, Luis Becker, Sandra Reitmaier, Maxim Bashkuev, Georg N. Duda, Hendrik Schmidt

**Affiliations:** 1 Center for Musculoskeletal Surgery, Charité – Universitätsmedizin Berlin, Charitéplatz 1, 10117 Berlin, Germany; 2 Julius Wolff Institute, Berlin Institute of Health at Charité – Universitätsmedizin Berlin, Augustenburger Platz 1, 13353 Berlin, Germany

**Keywords:** Low back pain, Movement, Physiotherapy, Conservative treatment, Chronic, Lumbar spine, Chronic pain, Lifestyle modification

## Abstract

Recent research has increasingly highlighted the role of movement behavior in the onset and persistence of low back pain (LBP). However, little is known about the lumbar spine’s movement patterns in daily life. This study investigated the number of lumbar movements in asymptomatic individuals and those with chronic LBP (lasting ≥ 12 weeks) over a 24-hour period. Lumbar movements were measured with the Epionics SPINE system. Movements ≥ 5° were grouped into movement sizes of > 15°, 10–15°, and 5–10°. Data were analyzed using the Student’s t-test, two-way analysis of variance, or the Pearson’s correlation coefficient. This study included 208 asymptomatic participants and 106 LBP participants. Participants with LBP exhibited a significantly lower number of both flexion/extension (15,564 ± 8,078 vs. 20,521 ± 7,160, *p* < 0.001) and rotation movements (4,724 ± 3,995 vs. 7,368 ± 4,223, *p* < 0.001). Females showed significantly more flexion/extension movements > 15° compared to males. Participants were significantly older than asymptomatic participants (40.3 ± 14.0 vs. 50.9 ± 13.9 years, *p* < 0.001). The correlation between age and the number of flexion/extension movements was weak in both the LBP (*r*=-0.290, *p* = 0.003) and the asymptomatic (*r*=-0.179, *p* = 0.010) groups. In summary, individuals with chronic LBP exhibit fewer lumbar spine movements than their asymptomatic counterparts, and distinct sex differences in movement patterns were observed, with females showing a different movement profile compared to males.

**Supplementary Information:**

The online version contains supplementary material available at 10.1038/s41598-025-04851-2.

## Introduction

Low back pain (LBP) is one of the leading causes of years lived with disability and affects at least 80% of adults at some point in their lives^1^. Accordingly, LBP is one of the most commonly treated conditions in primary care and due to the high rate of associated disability causes a significant burden not only on affected individuals and their families but on the whole socioeconomic system^2^. However, due to a lack of understanding regarding the underlying mechanisms of pain development and persistence therapeutic management of chronic LBP remains challenging.

In the presence of pain, spinal movement may change, which in the beginning is thought to be protective but in the long term may turn into a cause of further pain^3^. To date, the underlying mechanisms of these changes are poorly understood, which is why studies analyzing spinal motion in the context of chronic pain as well as the impact of movement adaptations on LBP have gained increasing attention. During a variety of tasks, it has been shown that LBP is associated with restricted spinal movement, excessive trunk muscle activity, and reduced movement variability, all of which may contribute to pain persistence and disability^4–7^. Furthermore, in the context of fear avoidance, individuals with LBP show restricted daily activities in an attempt to reduce any exposure to pain^4^. In the presence of LBP, participants are also likely to avoid painful activities, which may limit specific movements^8,9^.

In treating and managing LBP, maintaining an active lifestyle is associated with a reduction of disability and increase of quality of life^10,11^. Movement generally is thought to not only have an analgesic effect but also prevent pain from turning chronic^12,13^. However, at the same time exercise may in fact increase pain, indicating that the relationship between pain and movement is complex and far from understood^14^.

While the current literature emphasizes the importance of spinal movement and physical activity in both the prevention and treatment of LBP, little is known about normal spinal activity patterns, contributing to the wide variability in exercise interventions of LBP^15^. In 2014, Rohlmann et al. published the first study investigating spinal movements over a 24-hour period in asymptomatic individuals during daily activities^16^. Despite this, evidence linking reduced lumbar movement with the presence of LBP is still lacking. Thus, the present study aimed to quantify the number and distribution of spinal movements over 24 h in both asymptomatic individuals and those with LBP. We hypothesized that individuals with LBP exhibit a significantly lower number of spinal movements compared to asymptomatic individuals, with additional sex-specific differences in spinal movement patterns. Furthermore, given previous findings indicating that advancing age is associated with diminished lumbar range of motion, we also hypothesized that the number of lumbar movements decreased with increasing age^17,18^.

## Methods

### Participants

The study was approved by the institutional ethics committee (Ethikkommission Charité – Universitätsmedizin Berlin, registry numbers EA4/011/10, EA1/162/13). The reporting is in accordance with the Strengthening the Reporting of Observational Studies in Epidemiology (STROBE) guidelines^19^. We prospectively enrolled participants who had either no history of LBP in the preceding six months or chronic LBP, defined as pain persisting for at least twelve weeks^20^. Data for the asymptomatic cohort were collected between September 2010 and November 2011 and partially published by Rohlmann et al.^16^ In that study, results for movements in the sagittal plane were reported, while movements in the transverse plane were not analyzed. Data for participants with LBP were collected between September 2022 and March 2023. All asymptomatic and LBP participants provided written informed consent. Exclusion criteria for both groups included neurological symptoms, a body mass index (BMI) greater than 26 kg/m², and previous spinal surgery.

### Measurement system

Measurements were performed with the Epionics SPINE system (Epionics Medical GmbH, Potsdam, Germany), which consists of two flexible sensor strips, two tri-axial accelerometers, and a small storage box^16,21,22^. Each sensor strip has twelve predetermined 25-mm‐long segments, which measure the segment angles at a frequency of 50 Hz using strain‐gauge technology. The sensors’ orientation in relation to the earth’s gravitational field is measured by accelerometers at the bottom end of the sensor strips. Special hollow plasters were glued to the participants’ backs to the left and right of the spine at a mid-line-distance of 7.5 cm each before inserting the sensor strips. The posterior superior iliac spine was defined as the standard location for the most caudal sensor. The Epionics SPINE system has an accuracy of approximately ± 2 degrees when measuring lumbar spine angles. The sensor strip exhibits a high repeatability (ICC > 0.98), with test‐retest reliability ICCs of > 0.98, as well as a high accuracy, also compared to other measurement systems^21^. Furthermore, the Epionics SPINE system has previously been shown to have high sensitivity and specificity in recognizing movements in flexion/extension, and axial rotation^23^.

### Measurement protocol

The measurement protocol has been previously described in detail and is briefly summarized below^16^.

**Fig. 1:**
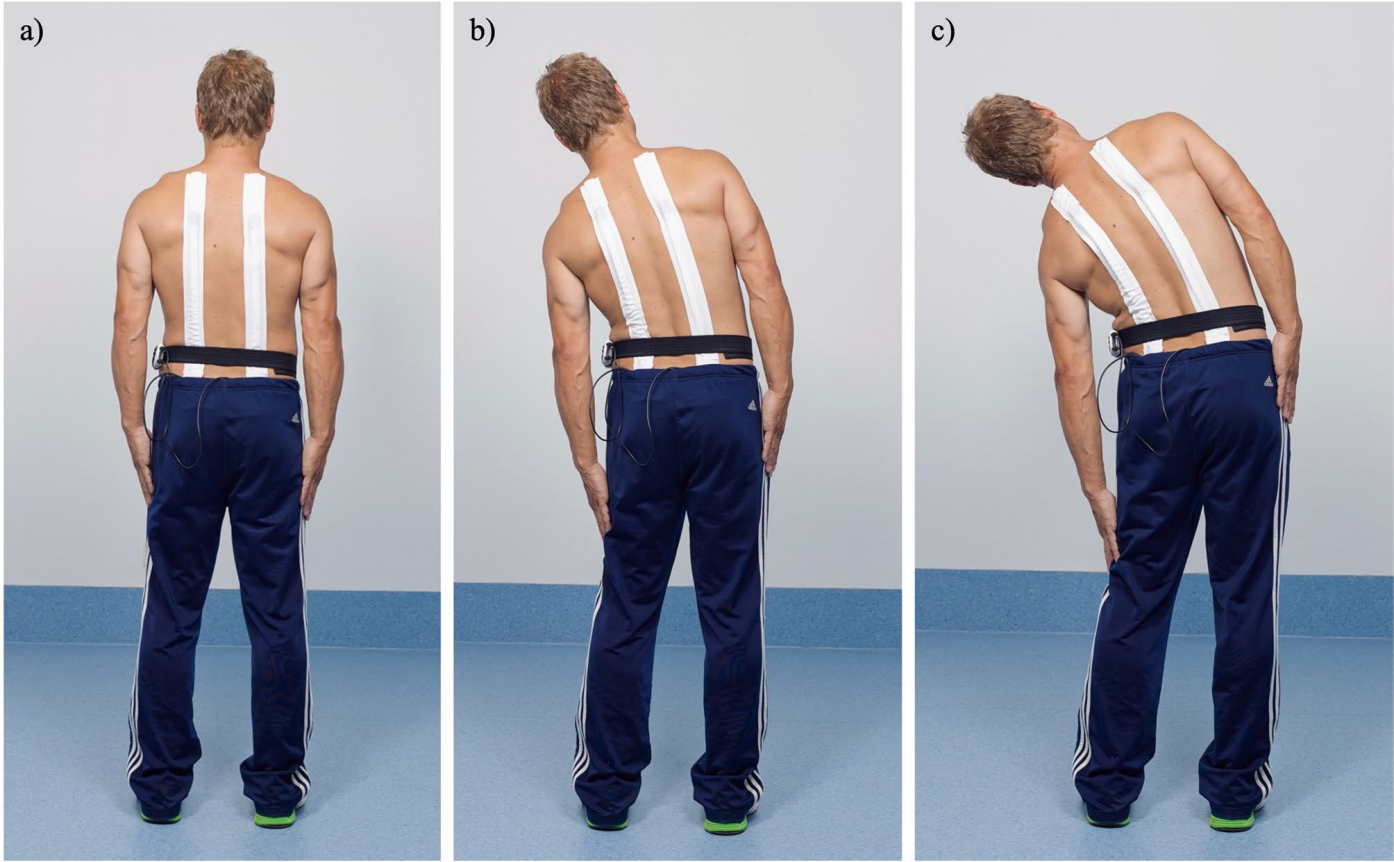
(**a**) The Epionics SPINE system affixed to a volunteer’s back in standing. The system consists of two flexible sensor strips utilizing strain-gauge sensors, tri-axial accelerometers, and a storage unit. (**b**, **c**) Lateral bending as part of the sequence of choreographed exercises.

Study participants were equipped with the Epionics SPINE system (Fig. 1) between 7 and 10 a.m. and instructed to perform a series of controlled movements in both the sagittal and transversal planes. The movement sequence began with a relaxed, upright standing posture, followed by maximal upper body flexion, extension, and left and right axial rotations, all performed with knees extended and arms in a relaxed, gravity-directed position. Each movement was repeated six times at the participants’ preferred pace to ensure natural movement patterns. To standardize performance, participants viewed an instructional video prior to the choreography, which demonstrated and explained each exercise.

After completing the short-term measurements and before leaving the lab, participants were instructed to maintain their regular daily routines while avoiding any unusual physical activities that could impact their natural lumbar postures. They were specifically advised not to shower during the 24-hour monitoring period to prevent any potential disruption to the Epionics SPINE system. After the 24-hour monitoring was completed, participants returned to the lab for the removal of the Epionics SPINE system. The recorded data were then uploaded and carefully inspected for completeness and quality. Any anomalies, such as device displacement or data gaps, were noted and addressed during data analysis.

### Data analysis

Data analysis was performed as previously described by Rohlmann et al.^16^ The thoracolumbar lordosis angle (LA) of the relaxed standing position was defined as a reference value and was determined by calculating the median LA of all six standing postures measured during the short choreography. The LA was defined as the sum of the six most caudal sensor strip segments averaged between left and right strips. Pure flexion and extension of the upper body was characterized by a symmetrical motion in the sagittal plane with almost identical readings at the left and right sensors. Here, the LA at each time frame was determined as the sum of the angles at associated sensor segments. Asymmetric motions of the upper body led to different LAs on the left and right sides of the back. Axial rotation was approximated as the rotation of a line segment connecting the cranial edges of the lordotic segments around the chord of the lordotic arc calculated as an average of the left and right stripes.

For determination of the total number of movements, exactly 24 h (4.32 million frames) of data were analyzed. An eighth-order low-pass Butterworth filter with a cut-off frequency of 5 Hz was used to filter the recorded raw data from the sensor strips and eliminate noise. Movements were counted if they were greater than 5° and were grouped into movement sizes of 5–10°, 10–15°, and > 15°. A movement considered the starting LA and was ended when a countermovement of > 5° was detected. Thus, a movement of -15° could be from a flexed posture of 45° to a less flexed posture of 30°. Based on a certain LA in the sagittal plane and a certain rotation angle in the transverse plane, a movement was counted separately as forward (+) / right rotation (+) and backward (-) / left rotation (-). Lying down was identified based on the accelerometers’ orientation and a reduction of variance in the accelerometer data. The longest period of lying down was defined as sleeping and was omitted from the analysis.

### Statistical analysis

Data were initially tested for normal distribution using the Kolmogorov-Smirnov test. For comparison of unpaired parametric parameters, the Student’s t-test was employed. To compare the analyzed groups regarding the number of distinct movements performed, a two-way between-subjects analysis of variance (ANOVA) was conducted, with factors including sex (male, female) and pain status (asymptomatic, low back pain [LBP]) using partial eta-squared to calculate effect sizes. Statistically significant main effects were further analyzed using independent samples t-tests. To analyze the correlation between age and the number of lumbar movements, Pearson’s correlation coefficient was calculated. A p-value of < 0.05 was considered statistically significant. All statistical analysis were performed using SPSS Version 27.

## Results

A total of 208 asymptomatic participants and 176 LBP participants were measured, of which 208 asymptomatic and 106 LBP participants fulfilled the BMI inclusion criteria. Demographic data for the two groups are shown in Table 1. LBP participants were significantly older (50.9 ± 13.6 vs. 40.3 ± 14.0 years, *p* < 0.001) compared to asymptomatic participants.

**Table 1:** Demographic data of included asymptomatic participants and patients with low back pain (LBP).

	Asymptomatic participants (*n* = 208)	LBP patients (*n* = 106)	*p*-value
Age (years)	40.3 ± 14.0	50.9 ± 13.6	< 0.001*
Gender (f: m)	115:93	63:43	0.483
BMI (kg/m^2^)	22.6 ± 2.0	23.1 ± 2.3	0.070

For age and body mass index (BMI), mean values with standard deviation are given. * indicates statistically significant differences.

**Fig. 2:**
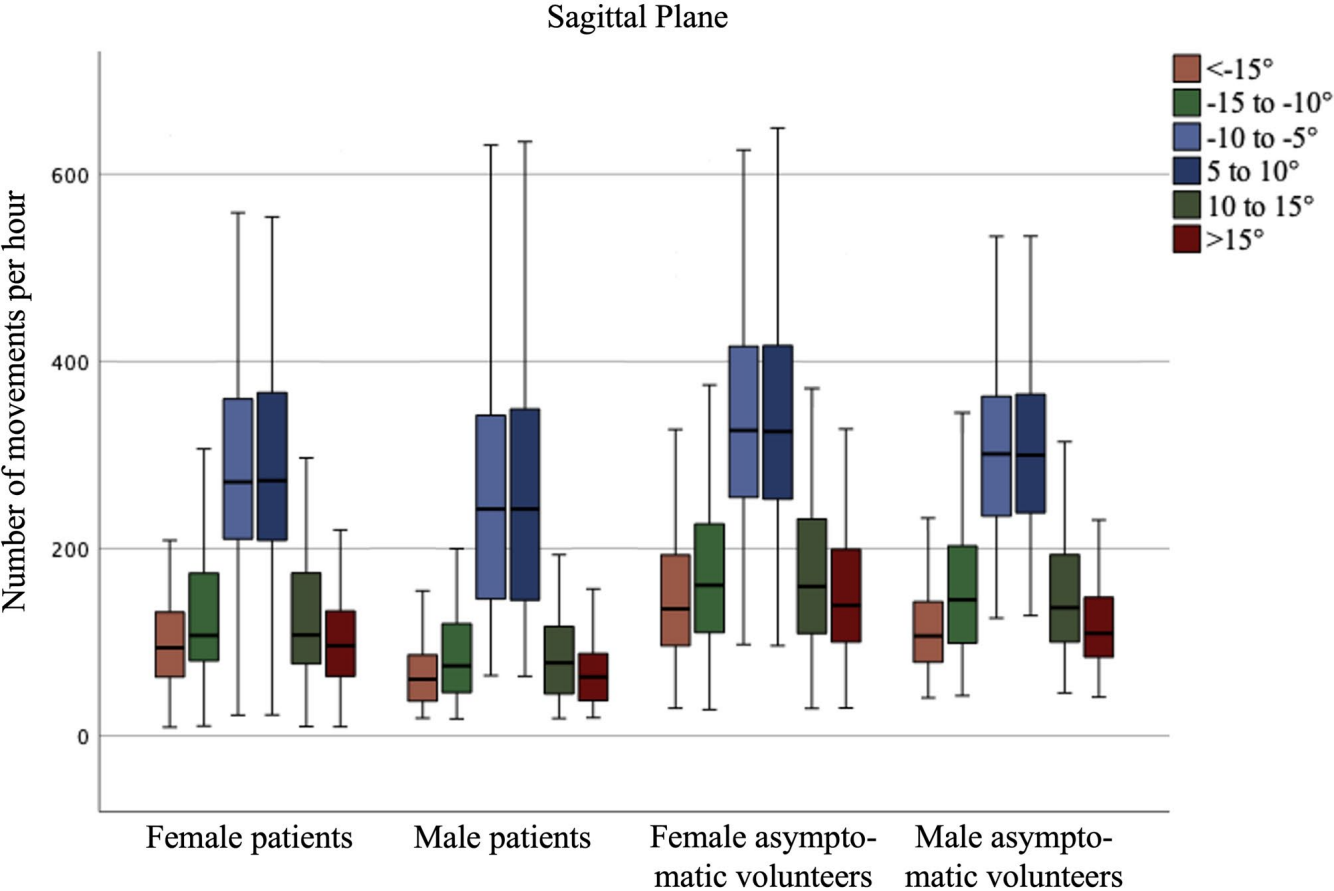
Average number of forward and backward movements per hour in the sagittal plane during the day (24 h minus sleeping time).

**Fig. 3:**
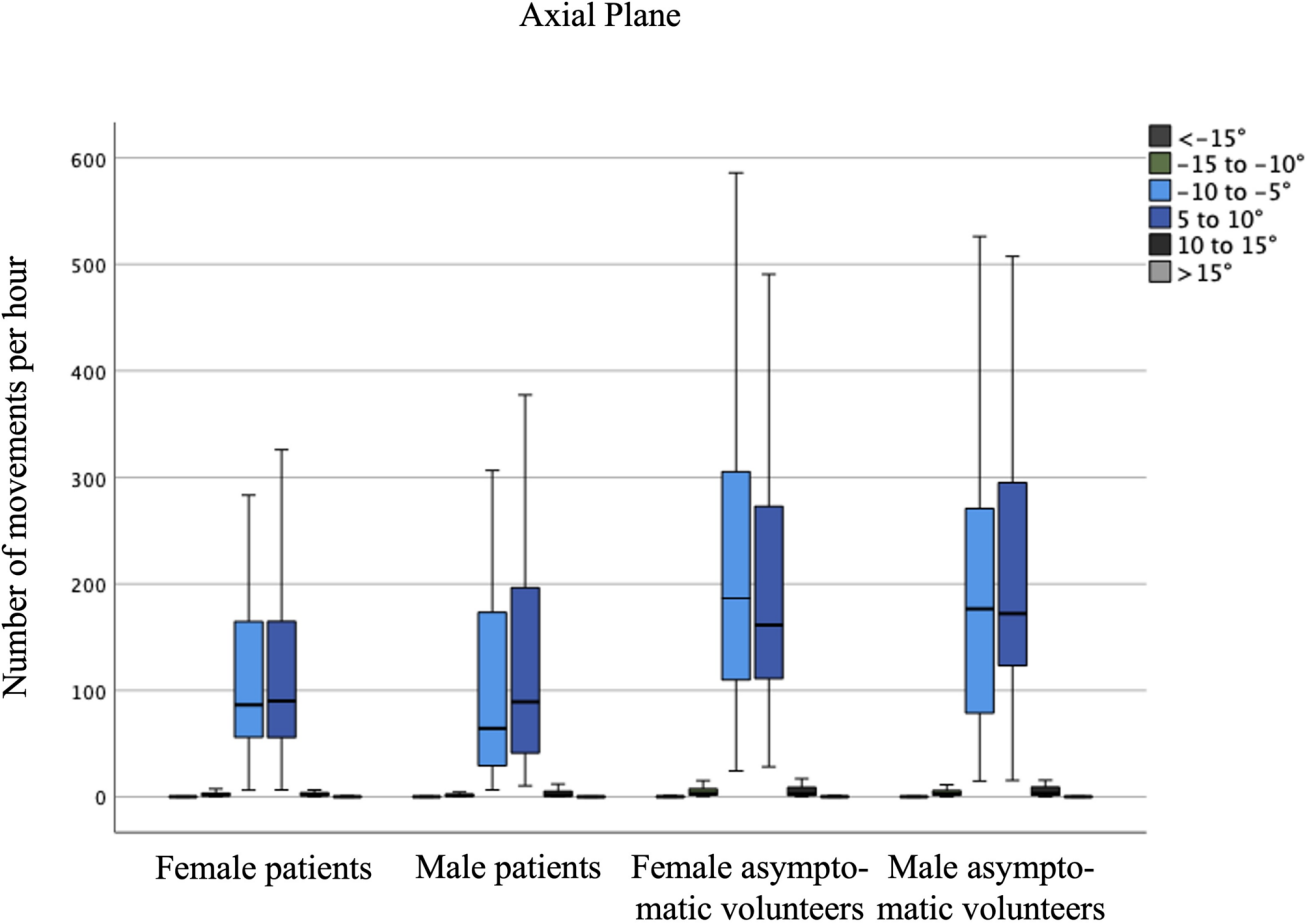
Average number of lumbar axial rotation movements per hour during the day (24 h minus sleeping time).

There was no significant difference between forward (+) and backward (-) movements, nor between right (+) and left (-) rotations, as each movement was counted independently from the starting position. Consequently, the frequency of executed movements within the defined movement amplitudes followed a normal distribution for each study group, as illustrated in Figs. 2 and 3. With 15,564 ± 8,078 movements in total, LBP participants performed significantly fewer (*p* < 0.001) forward and backward movements than asymptomatic participants with 20,521 ± 7,160 during the day. A similar trend was observed for axial rotations; LBP participants completed 4,724 ± 3,995 total right and left rotations, which was significantly fewer (*p* < 0.001) than the 7,368 ± 4,223 rotations performed by asymptomatic participants. In both groups, females showed significantly more spinal movements than males. The number of movements during the day per hour for each movement groups is presented in Table 2. The results for each movement group in the sagittal and axial planes are presented in Figs. 2 and 3, respectively.

**Table 2:** Number of lumbar spinal movements per hour in the sagittal (flexion/extension) and axial (rotation) planes during the day.

	Asymptomatic participants		LBP patients	
	Females (*n* = 115)	Males (*n* = 93)	Females (*n* = 63)	Males (*n* = 43)
Forward (+) and backward (-) movements				
Total	1,364 ± 478	1,181 ± 386	1,072 ± 507	827 ± 470
< -15°	155 ± 87	118 ± 55	111 ± 94	69 ± 42
−15° to −10°	182 ± 98	161 ± 81	135 ± 88	91 ± 62
−10° to −5°	345 ± 120	312 ± 104	290 ± 118	253 ± 147
5° to 10°	344 ± 121	313 ± 105	288 ± 117	252 ± 146
10° to 15°	181 ± 94	157 ± 79	135 ± 87	92 ± 61
> 15°	157 ± 86	121 ± 55	113 ± 94	71 ± 43
Right (+) & left (-) axial rotations				
Total	440 ± 251	419 ± 234	273 ± 208	270 ± 275
< −15°	0 ± 1	0 ± 1	1 ± 1	1 ± 1
−15° to −10°	8 ± 13	8 ± 13	4 ± 10	3 ± 6
−10° to −5°	215 ± 133	193 ± 124	130 ± 118	115 ± 129
5° to 10°	205 ± 131	206 ± 115	131 ± 117	143 ± 134
10° to 15°	11 ± 26	12 ± 23	7 ± 15	8 ± 24
> 15°	0 ± 1	0.3 ± 1	1 ± 5	1 ± 1

In the two-way ANOVA, no significant interaction effect was observed between the variables pain status and sex across any movement groups. However, significant main effects were identified for both sex and pain status across all movement groups in the sagittal plane. In the axial plane, a significant main effect of pain status was observed for all movement groups, except for movements exceeding 15° or less than − 15° (Table 3).

**Table 3:** Results of the two-way ANOVA analyzing the influence of the factors sex and pain status regarding the number of distinct movements performed.

	Sex			Pain status		
	Mean difference	p-value	Partial Eta^2^	Mean difference	p-value	Partial Eta^2^
Forward (+) and backward (-) movements						
<−15°	40 ± 9	< 0.001*	0.057	46 ± 9	< 0.001*	0.077
−15° to −10°	33 ± 11	0.002*	0.030	59 ± 11	< 0.001*	0.091
−10° to −5°	35 ± 14	0.017*	0.018	57 ± 14	< 0.001*	0.048
5° to 10°	34 ± 14	0.020*	0.017	59 ± 14	< 0.001*	0.051
10 to 15 °	34 ± 10	0.001*	0.034	56 ± 10	< 0.001*	0.088
> 15°	39 ± 9	< 0.001*	0.057	47 ± 9	< 0.001*	0.079
Right (+) & left (-) axial rotations						
<−15°	3 ± 2	0.156	0.006	4 ± 2	0.991	0.000
−15° to −10°	0 ± 8	0.977	0.000	23 ± 8	0.004*	0.027
−10° to −5°	14 ± 17	0.404	0.002	98 ± 17	< 0.001*	0.095
5° to 10°	15 ± 17	0.398	0.002	98 ± 17	< 0.001*	0.095
10 to 15 °	0 ± 8	0.999	0.000	23 ± 8	0.005*	0.026
> 15°	3 ± 2	0.151	0.007	4 ± 2	0.990	0.000

Positive mean differences represent a greater number of movements in females than males and a greater number of movements in healthy participants. * indicates statistically significant differences.

For flexion/extension movements, a weak but statistically significant negative correlation was observed between age and the number of movements in both the LBP group (*r*=-0.290, *p* = 0.003) and the asymptomatic group (*r*=-0.179, *p* = 0.010). Similarly, for axial rotation movements, a weak but significant negative correlation between age and the number of movements was found in the asymptomatic group (*r*=-0.198, *p* = 0.004) but not in the LBP group (*r*=-0.133, *p* = 0.173) group.

## Discussion

Even though both the prevention and adequate treatment of LBP are of high clinical relevance, to date little is known about spinal movement behavior across a day and across daily activities. Therefore, this study aimed to investigate the quantity of lumbar movement patterns in asymptomatic participants and in participants with LBP during daily life. We show that overall, participants with LBP perform a significantly reduced number of lumbar movements both in the sagittal and in the axial plane compared to asymptomatic participants. Both sex and pain status showed significant main effects on the number of lumbar movements in the sagittal plain, while in the axial plane only pain status showed significant main effects on the number of lumbar movements.

In explaining the relationship between pain and motion adaptation, multiple theories have been proposed. Early theories postulated that in the presence of pain, muscle activity increases which may cause ischemia and subsequent stimulation of nociceptive afferents^24^. Other theories emphasized the role of fear avoidance and cognitive-emotional mechanisms in explaining reduced movement and function as a result of pain^25^. It seems undeniable that the underlying pathophysiology is complex and not only stems from a combination of proposed mechanisms but differs between individuals. Therefore, more recent theories regarding this interplay are more comprehensive and take into account that movement adaptations may be both cause and effect of pain, are generally aimed at protecting the affected body part from further pain, potentially have long-term consequences, and may be influenced by a variety of factors including psychosocial ones^3^. While in the context of pain, movement adaption in most cases is an attempt of the body to prevent further damage or pain and in that capacity may initially benefit the patient, in the long term these changes may become part of the problem and cause further deterioration or persistency of symptoms.

Thus, adaptations of movement behavior need to be taken into consideration in developing treatment plans for this highly challenging patient cohort. In the literature, there appears to be a general consensus that patients with non-specific LBP should maintain normal activities and in case of chronic LBP be prescribed exercise therapy^26^. However, published guidelines show a significant variance in recommendations regarding the type of exercise programs and the mode of delivery. This inconsistency is due to a lack of understanding regarding the detailed interplay of spinal movement, physical activity, and pain development or chronicity. Furthermore, the paradox relationship between physical activity and pain needs to be taken into consideration: even though movement is part of the treatment of LBP, it may also provoke pain. Moreover, it remains debatable whether the adapted movement behavior in fact continuously contributes to persistent pain and thus needs to be addressed as part of any treatment strategy. The question remains which factors of movement need to be primarily addressed when treating pain and associated disability. Is it as simple as suggesting the patient to stay active? How important is individual movement behavior? Do patients need to simply move more, or do they need to move differently? In this context, previous studies have shown that cognitive functional therapy, which is a psychologically informed approach addressing pain-provocative movement patterns such as movement avoidance, lead to improved clinical results compared with usual care^27,28^.

Despite these open questions regarding motion behavior, the quantity of performed lumbar movements during daily life to date has not been analyzed in depth. In particular, the studies investigating movement in a quantitative fashion mostly look at the performance of a certain amount of physical activity rather than specific spinal movement patterns^29^. To gain a more detailed understanding of the pathogenesis of LBP, it seems necessary to not only describe the movement behavior of healthy individuals but more importantly to investigate any differences that may be present in people with LBP. In this regard, our results show that individuals with LBP perform a significantly reduced overall amount of lumbar spinal movements compared with asymptomatic individuals in both the sagittal and axial planes. This difference was significant for small, medium, and large movements in the sagittal plane, which is in line with previous reports showing that individuals with LBP have a reduced sagittal lumbar amplitude^30^. For axial rotation movements, on the other hand, we only found significant differences for small movements of over 10°. This is most likely due to the overall few large rotational movements performed.

While we were also able to show a significant negative correlation between age and the number of lumbar spinal movements for both the asymptomatic cohort and LBP participants as well, this correlation was weak and was potentially driven by our study’s sample size. Nevertheless, considering the above-mentioned literature, it seems likely that with increasing age, spinal movement is reduced which facilitates both pain development and pain persistency. This underlines the complex relationship between motion behavior and pain development as the question remains whether with increasing age, spinal movement is reduced as a natural process and this is the cause for LBP development or whether with increasing age there is an increasing prevalence of LBP and this is the cause for a reduction in lumbar movement.

Moreover, we found sex to significantly influence the number of lumbar movements with females performing significantly more movements in the sagittal plane compared with males. This is counterintuitive as females are more commonly affected by LBP and it is assumed that a higher number of movements is both protective regarding pain development and should be part of LBP treatment^31^. While previous studies have found an increased spinal range of motion and velocity to be associated with improved pain, this has not been shown for the amount of spinal movement^5^. Moreover, it remains unclear whether movement changes result in pain reduction or whether pain reduction results in less protective movement^32^.

In the past, in the presence of LBP or sciatica bed rest was a common treatment approach^11^. However, since then it has been proposed that prolonged inactivity is potentially harmful which is why by the end of last century, a more active approach was introduced in the treatment of LBP^26^. While in pain-free individuals it has been shown that exercise induces pain inhibition, there is a lack of high-quality evidence for this effect in patients with chronic pain^33,34^. However, previous studies show an association between a less active lifestyle and greater pain and disability^35^. Regarding the role of movement for the general health, benefits also include the re-engagement in social activities and thus the psychosocial impact needs to be taken into consideration as well^36^.

Some limitations of our study need to be discussed. As this study represents the first long-term measurements of spinal posture using continuous 24-hour monitoring, there were no prior data available to conduct a traditional sample size calculation. The exploratory nature of our study, aimed at establishing the variability of lumbar postures in daily life, made it difficult to predict effect sizes or patterns from existing literature. Therefore, we focused on gathering a representative sample while ensuring practical constraints related to the novel nature of the methodology. Moving forward, we anticipate that the data gathered from this study will be invaluable for informing future sample size calculations in similar research. Spinal movements were measured on the back and not directly in the spine. However, previous studies have shown a correlation between posture and motion measured on the back and the spine^37,38^ and Suter et al. found high agreement between the Epionics SPINE system and a motion capture system in measuring lumbar curvature angles^39^. Data on the participants’ profession was not collected which is why an analysis regarding differences between physical and intellectual work was not possible. Furthermore, our study design was observational, which is why we cannot make any statements regarding the results’ causality.

## Conclusion

Our findings indicate that individuals with LBP perform significantly reduced movements in both the sagittal and axial plane over a 24-hour period compared to an asymptomatic cohort. However, the causal relationship between altered movement behavior and the onset and chronicity development of pain remains ambiguous, as does the correlation with pain intensity. While an association between movement alterations and pain appears substantial, necessitating careful consideration in the planning of conservative treatment approaches, it is essential to conduct a more in-depth analysis of this relationship. Such analysis should focus on the intricate interplay among movement, cognition, psychological factors, and pain. Additionally, future research should account for variations in occupational demands and levels of physical activity to enhance our understanding of these dynamics.

## Electronic supplementary material

Below is the link to the electronic supplementary material.

Supplementary Material 1

## Data Availability

The datasets generated during and/or analysed during the current study are available in the supplementary files.
